# Versatile Recombinant SUMOylation System for the Production of SUMO-Modified Protein

**DOI:** 10.1371/journal.pone.0102157

**Published:** 2014-07-09

**Authors:** Alain R. Weber, David Schuermann, Primo Schär

**Affiliations:** Department of Biomedicine, University of Basel, Basel, Switzerland; Institute of Molecular Genetics IMG-CNR, Italy

## Abstract

Posttranslational modification by small ubiquitin-like modifiers (SUMO) is being associated with a growing number of regulatory functions in diverse cellular processes. The biochemical investigation into the underlying molecular mechanisms, however, has been lagging behind due to the difficulty to generate sufficient amounts of recombinant SUMOylated proteins. Here, we present two newly designed two-component vector systems for the expression and purification of SUMO-modified target proteins in *Escherichia coli*. One system consists of a vector for SUMO conjugation, expressing human SUMO-activating (SAE1/SAE2) and conjugating (Ubc9) enzymes together with His_6_-tagged SUMO1, 2 or 3, that can be combined with commonly used expression constructs for any gene of interest. To facilitate SUMOylation of targets normally requiring a SUMO-E3 ligase for efficient modification, a second system is designed to express the target protein as a fusion with the human SUMO-conjugating enzyme Ubc9, thus compensating the absence of a potential SUMO ligase. We demonstrate the proficiency of these systems by SUMOylation of two DNA repair proteins, the thymine DNA glycosylase (TDG) and XRCC1, and describe purification schemes for SUMOylated proteins in native and active form. This SUMO toolbox facilitates “in-cell” and “in-extract” production and purification of recombinant SUMO-modified target proteins for functional and structural analysis.

## Introduction

**Figure 1:**
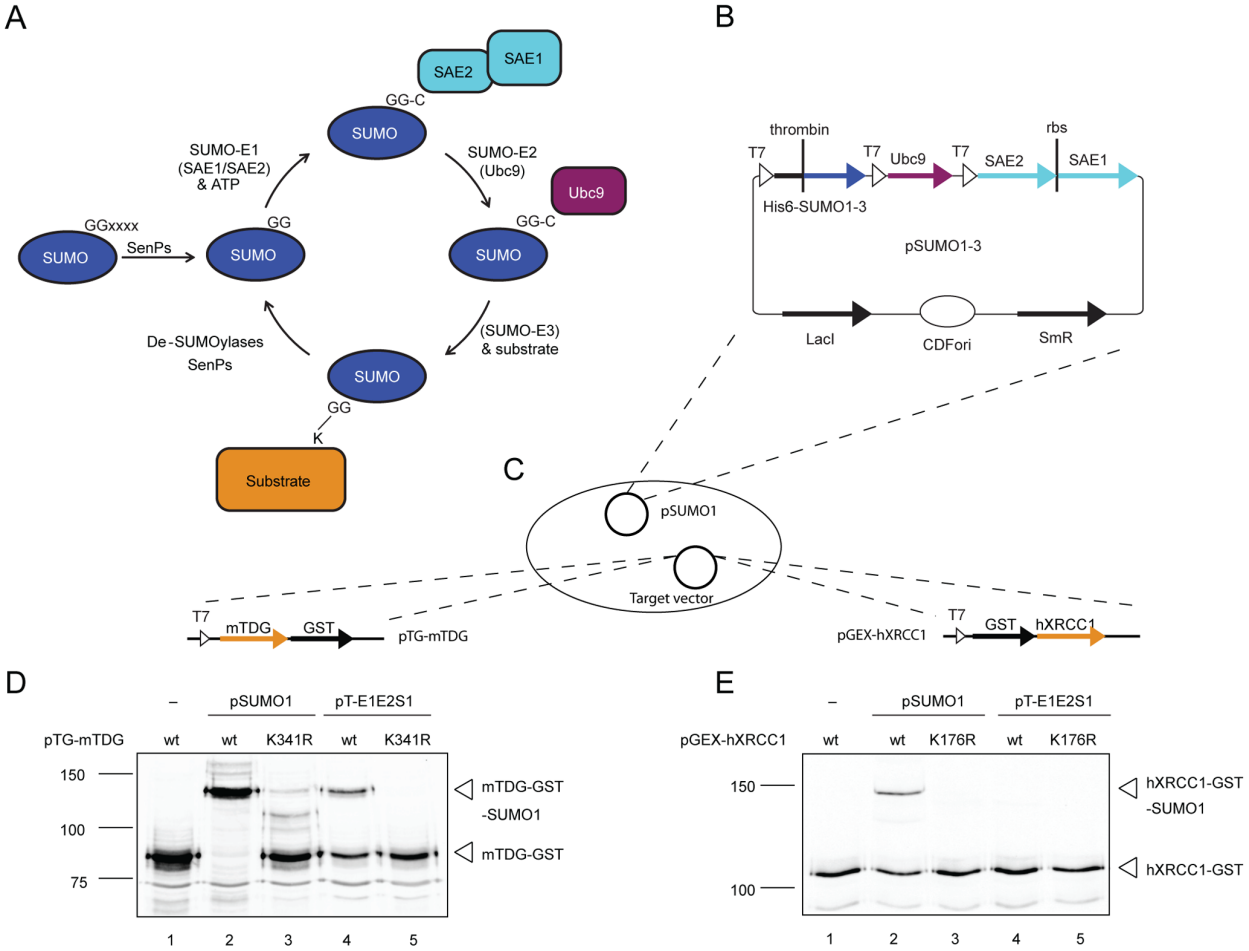
The pSUMO-based SUMOylation system modifies proteins in *E.coli*. (**A**) Scheme of the *in vivo* SUMO maturation, SUMO conjugation and deconjugation process (for detailed description, see “Introduction”). (**B**) pSUMO vectors containing the humanized SUMOylation system consisting of N-terminally His_6_-tagged SUMO1, 2 or 3, the SUMO-conjugating enzyme E2 (Ubc9) and both subunits of the SUMO-activating enzyme (SAE1 and SAE2) as a cistronic expression unit with an internal ribosomal binding site (rbs). Expression of the respective cDNAs is under the control of a lac-repressor (LacI) regulated T7 promoter. (**C**) Scheme of the experimental setup of the pSUMO-based in-cell SUMO conjugation. *E.coli* BL21 cells were used containing pSUMO1 in combination with pTG-mTDG or pGEX-hXRCC1 plasmids were used for the co-expression of the complete SUMO system with C- and N-terminally GST-tagged mTDG and hXRCC1, respectively. Immunoblot analyses of mTDG (**D**) and hXRCC1 (**E**) SUMOylation in *E.coli* cells, expressing the SUMO target and the SUMO system from either the pSUMO1 or the pT-E1E2S1 plasmid (250 µM IPTG at 25°C for 2 h). Co-expression of target proteins mutated in the SUMO acceptor sites of mTDG (K341R) and hXRCC1 (K176R) were included to assess the specificity of the SUMOylation system.

Posttranslational modification by ubiquitin-like polypeptides, so-called UBLs, affects a large number of proteins, thereby regulating a variety of cellular processes [1], [2]. The SUMO (small ubiquitin-like modifier) peptides represent a prominent subfamily of the UBLs and exist in four different isoforms (SUMO1, SUMO2, SUMO3 and SUMO4) in mammalian cells, each encoded by a different gene. Although these SUMOs differ to some extent in their amino acid sequences – SUMO2 and SUMO3 share sequence identity of 97% with each other and about 50% with SUMO1 – they all show high 3D-structural resemblance [3]–[6]. SUMOs, as all UBLs, are attached to their target proteins by a sequence of enzymatic reactions resembling those of ubiquitin conjugation [7], [8], involving a heterodimeric activating enzyme E1 (SAE1/SAE2), a single conjugating enzyme E2 (Ubc9) and, in some cases, an E3 protein ligase (Figure 1A). SUMO itself is first synthesized as a precursor peptide that is then trimmed by a SUMO-specific isopeptidase (sentrin-specific proteases; SenPs) to expose an internal glycine-glycine (GG) motif at the C-terminus. The carboxyl group of this mature SUMO peptide is then linked via a thioester to a cysteine residue in SAE2 in an ATP-dependent manner [9]. Subsequently, the activated SUMO is transferred to a cysteine residue of the SUMO-conjugating enzyme Ubc9 [10]. Ubc9 can recognize substrate proteins directly [11] and catalyze the formation of a peptide bond involving the C-terminal carboxyl group of SUMO and an ε-amino group of a target lysine within the SUMOylation consensus motif ΨKxE (Ψ, hydrophobic residue; x, any residue) of the substrate protein [12]. Often, however, SUMO conjugation is additionally promoted by SUMO-E3 ligases, which act as substrate-specific adapters (Figure 1A).

SUMO modification concerns a wide spectrum of target proteins, implicating functions in a variety of vital biological processes such as the cellular response to DNA replication stress, the repair of DNA damage, the regulation of gene expression and epigenetic DNA and histone modifications [2], [5], [13]. Although research into protein SUMOylation has identified a large number of targets over recent years, there is limited insight into the functional consequences of the modification. Investigations into the immediate biochemical and structural impact of SUMO modification have been a challenge due to difficulties in producing SUMO-modified proteins in sufficient amounts and homogeneity. Enrichment of native endogenous SUMOylated proteins by cell fractionation is generally limited by the low abundance of such proteins and the action of efficient SUMO proteases [14]. *In vitro* modification of enriched target proteins with recombinant SUMOylation enzymes is a more promising approach, typically yielding mixtures of modified and unmodified target proteins contaminated with the SUMOylation enzymes, hence requiring subsequent purification steps [15], [16]. Also, co-expression of SUMO targets with SUMO1 and SUMO-activating and -conjugating enzymes of different origin (human, mouse, *Xenopus laevis*) in *E.coli* was shown to produce SUMO-modified protein [17]–[19], but in our experience, the yields of specifically modified proteins were often poor, impeding efficient purification and subsequent biochemical analysis.

To streamline the production of homogeneously SUMOylated recombinant proteins for biochemical and structural studies, we set out to establish an optimized and versatile SUMOylation system, coupling efficient SUMO-conjugation with affinity purification of modified target proteins. We designed and experimentally validated two alternative two-component vector systems for simultaneous expression of mature SUMO1, 2 or 3 polypeptides, SUMO-E1, SUMO-E2 and a target protein of interest in *E.coli*. In contrast to previous approaches [18]–[20], we used SUMOylation enzymes of human origin only, physically separated the SAE1 and SAE2 E1 subunits and added a protease-cleavable His_6_-tag to the SUMOs to facilitate purification of modified protein. To overcome a possible rate limitation by the absence of an appropriate SUMO-E3 ligase in the *E.coli* system, one system is designed to express the target protein as fusion to the SUMO-conjugating enzyme Ubc9, a strategy that was successfully applied for SUMOylation of ectopically expressed p53 and STAT1 in HEK293, HeLa, COS-7 and CHO cells [21], [22]. We evaluated these newly developed vector systems with the DNA base excision repair (BER) enzymes thymine DNA glycosylase (TDG) and XRCC1. TDG is a well-studied SUMO target [23]; SUMO modification of TDG was shown to effect conformational changes that promote enzymatic turnover [24] and may also regulate the subcellular localization [25]. XRCC1 acts as central scaffold factor in BER [26] and was identified as a putative SUMO target in an *in vitro* screening approach. It was found SUMOylated in HeLa cells following heat shock treatment [27], [28], but the function of this modification remains to be elucidated. We assessed the efficiency and specificity of SUMO modification of these proteins in our recombinant systems as well as the proficiency of the purification procedure to generate biologically active SUMO proteins. Finally, we provide guidance to optimize the experimental setup and conditions for the SUMOylation of any target protein in bacteria, discussing the use of the different SUMOylation vectors and expression strategies for “in-cell” or “in-extract” SUMOylation.

## Materials and Methods

### Vector construction

The plasmids with the complete SUMO system (pSUMO1-3), the SUMO-activating (pSA1-3) and the SUMO-conjugating (pSC-PreE2/IntE2) plasmids were assembled by standard cloning methods based on PCR amplification with adaptor oligonucleotides providing suitable restriction sites. The plasmid DNA, vector sequences and maps are available from Addgene (http://www.addgene.org), plasmid ID 52258-52284. The cDNAs of the human SUMOylation components SAE1, SAE2, Ubc9 and SUMO1-3 were amplified from pGEX-based bacterial expression vectors kindly provided by R. Hay and M. Hottiger. pSUMO1-3, pSA1-3 and pSC-PreE2/IntE2 vectors are based on pCDFDuet-1 (Novagen) and pTXB3 (New England BioLabs), respectively. The cDNAs of the SUMO target proteins were amplified by adaptor PCR and introduced into the NcoI and EcoRI site (hXRCC1, hTDG) or NcoI and XhoI (mTDG) of the pSC-IntE2/PreE2 vectors. The consensus SUMOylation motif (VKEE) was deleted by site-directed mutagenesis in hTDG (K330A), mTDG (K341R), hXRCC1 (K176R).

### Recombinant protein expression, in-cell SUMOylation and cell lysis

The expression vectors were introduced into *E.coli* BL21(DE3) cells by electroporation. Overnight pre-cultures were diluted with fresh pre-warmed LB medium and grown at 30°C to OD_600_ levels as indicated. Cultures were grown under selective pressure using either 100 mg/L of Ampicillin or 50 mg/L of Streptomycin for single plasmid expressions and half the concentration of each antibiotic when co-expressing two plasmids. Protein expression was induced by the addition of the isopropyl-β-D-thiogalactose (IPTG) to the final concentrations as specified in the results and cultures were further incubated as indicated. Finally, cells were harvested by centrifugation and soluble protein fractions were extracted by sonication in lysis buffer (50 mM Na-phosphate buffer pH 7.5, 300 mM NaCl, 10% glycerol, 0.1% Tween-20, 1 mM DTT, 1 mM PMSF), if not stated otherwise. Crude lysates were then cleared by centrifugation with >20'000 g at 4°C for 30 min.

### Purification of SUMOylated protein

Small-scale protein preparations were performed with Glutathione sepharose HP (GE Healthcare) or cOmplete His-tag purification (Roche Applied Science) resins. Cleared lysates were incubated with 100 µL resin in lysis buffer at 4°C for 3 h, prior to loading onto gravity flow columns (BioRad). Unbound proteins were washed out with 20 and 10 bed volumes of lysis buffer with 0.3 and 1 M NaCl, respectively. After a final wash step with 10 bed volumes of lysis buffer, bound proteins were eluted by the addition of 250 mM imidazole or 10 mM reduced glutathione to the lysis buffer. Large-scale protein purification was carried out on an ÄKTA purifier 10 system using pre-packed columns (GE Healthcare). To enrich for SUMOylated proteins, cell lysates were loaded onto a 5 mL HisTrap crude FF column (GE Healthcare), washed with an imidazole gradient from 0 to 40 mM over 2 column volumes (CV) and bound proteins were eluted with 10 CV lysis buffer containing 400 mM imidazole. Peak fractions were pooled and dialyzed 3 times for 30 min against 300 mL GST loading buffer (50 mM Na-phosphate buffer pH 7.5, 500 mM NaCl, 15% glycerol, 0.1% Tween-20, 1 mM DTT, 0.5 mM PMSF) and loaded on a 1 mL GSTrap HP column (GE Healthcare). Unbound protein was washed out by a NaCl gradient from 0.5 to 1 M over 10 CV. Subsequently, bound target protein was released from the GST-Ubc9 moiety either by induced self-splicing at 4°C in cleavage buffer (50 mM Tris-HCl pH 8, 500 mM NaCl, 15% glycerol, 0.1% Tween-20, 0.1 mM PMSF, 50 mM DTT) for 16 h or by the application of 80 U of PreScission protease (GE Healthcare) according to the manufacturer's instructions. Cleaved protein was eluted, dialyzed 3 times for 30 min against 300 mL storage buffer (20 mM Tris-HCl pH 8, 50 mM NaCl, 10% glycerol, 1 mM DTT), snap-frozen and stored at −80°C.

### Analytical gel electrophoresis, western blotting and protein detection

Protein fractions were analyzed by standard SDS-polyacrylamide gel electrophoresis (SDS-PAGE) followed by Coomassie blue staining or by immunoblotting using the Odyssey imaging system (LI-COR Biosciences) or chemiluminescence (WesternBright ECL, Advansta) according to the provider's protocol. Antibodies were diluted in 5% non-fat dry milk TBS (100 mM Tris-HCl pH 8, 150 mM NaCl) supplemented with 0.2% Tween-20: human TDG, rabbit polyclonal ab 141 (raised against recombinant full-length hTDG), 1∶5'000; mouse TDG, rabbit polyclonal ab L58 (raised against recombinant full-length mTDG), 1∶5'000; XRCC1, rabbit polyclonal ab (Sigma-Aldrich X0629), 1∶2'000 and mouse monoclonal ab (33-2-5; Abcam ab1838), 1∶1'000; SUMO1, mouse monoclonal α-GMP1 ab (21C7; Life Technologies 33-2400), 1∶1'000 and rabbit polyclonal α-SUMO1 ab (Sigma-Aldrich S8070) 1∶1'000.

### Base release assay

A 60 bp heteroduplex DNA containing a G·U mismatch was prepared by annealing an unlabeled oligonucleotide (5′-TAGACATTGC CCTCGAGGTA CCATGGATCC GATGTCGACC TCAAACCTAG ACGAATTCCG-3′) to a 5′-fluorescein-labeled uracil-containing oligonucleotide (5′-ATCTGTAACG GGAGCTCCAT GGTACCTAGG CTACAGUTGG AGTTTGGATC TGCTTAAGGC-3′) by heating to 95°C for 5 min and gradual cooling to 25°C with a rate of 0.02°C/s.

Reactions were carried out in 20 µL nicking buffer (50 mM Tris-HCl pH 8, 1 mM EDTA, 1 mM DTT, 0.1 mg/mL BSA) containing 5 pmol of substrate DNA and 0.5 pmol of TDG protein, unless stated otherwise, at 37°C for the indicated time periods. AP-sites were then chemically cleaved by the addition of 2 µL of 1 M NaOH and boiling at 99°C for 10 min. DNA was precipitated overnight at −20°C after adding 2.2 µL of 3 M Na acetate pH 5.2, 0.5 µL yeast tRNA (10 mg/mL) and 67.5 µL ethanol. Subsequently, DNA was pelleted by centrifugation, washed with 70% ethanol, air-dried for 10 min, resuspended in 10 µL gel loading buffer (1×TBE, 90% formamide), heated at 99°C for 5 min and loaded on a 15% denaturing polyacrylamide gel (8 M urea, 1× TBE) for analysis. Gels were run at 13 V/cm for 30 min and labelled DNA was detected using the blue fluorescence mode of the Typhoon 9400 (GE Healthcare) and analyzed quantitatively by ImageQuant TL software (v7.0, GE Healthcare).

### SUMOylation and de-SUMOylation assays

*In vitro* SUMOylation with purified recombinant protein was carried out in 50 µL SUMOylation buffer (50 mM Tris-HCl pH 8, 50 mM NaCl, 10% glycerol, 0.5 mM DTT, 5 mM MgCl_2_, 5 mM ATP), containing 80 pmol SUMO1, 16 pmol Ubc9, 4 pmol SAE1/SAE2 and 16 pmol target protein. Reactions were incubated at 30°C for 30 min. De-SUMOylation was carried out in SenP2 buffer (25 mM Tris-HCl pH 8, 150 mM NaCl, 2 mM DTT, 0.1% Tween-20) using an excess amount of SenP2 protease. The reaction mixture was incubated at RT for 30 min.

For in-extract SUMOylation, cells expressing pSUMO1, pSA1 and target vectors were lysed in SUMOylation buffer without ATP and mixed at indicated volume ratios. SUMOylation was triggered by the addition of ATP to a final concentration of 5 mM and reaction mixtures were incubated at 30°C for 1 h.

## Results

### Recombinant human SUMOylation system modifies target proteins in *E.coli*

To provide a humanized SUMO-E1/E2 conjugation system for modification of target proteins in a recombinant bacterial expression setup, we constructed a series of CDFori-based vectors (pSUMO1-3), expressing the SUMO-E1 and -E2 enzymes as well as the mature SUMO1, 2 or 3 polypeptides (C-terminal GG motif) under the control of the phage T7 promoter (Figure 1B). Unique to this system is that it bases on human proteins only, expresses the heterodimeric SAE1-SAE2 complex (SUMO-E1) from a bi-cistronic unit and provides SUMO polypeptides with an N-terminal His_6_-tag separated by a thrombin cleavage site, facilitating the enrichment of modified proteins by affinity chromatography and the removal of the affinity tag.

We validated the functionality of the system by co-expression with an established and a postulated SUMOylation target of the BER pathway, TDG and XRCC1. To ensure a stable maintenance of the pSUMO1 and the target expression constructs in *E.coli*, we chose pMB1ori-based plasmids for the expression of the mouse TDG (mTDG) and the human XRCC1 (hXRCC1) (Figure 1C). First, we compared our pSUMO1 with the previously published pT-E1E2S1 vector [18] for the efficiency to produce SUMO1-conjugated C-terminally GST-tagged mTDG (pTG-mTDG) and N-terminally GST-tagged hXRCC1 (pGEX-hXRCC1) when co-expressed with the recombinant SUMO system in *E.coli* BL21(DE3) cells. Under the applied experimental conditions, we observed a significantly higher efficiency of SUMO1 modification with the newly designed pSUMO1 vector for both substrates, yielding nearly 100% SUMOylated mTDG and about 20% SUMOylated hXRCC1 (Figure 1D and E, compare lanes 2 and 4). The highly efficient modification in the presence of pSUMO1 may generate some unspecific SUMO conjugation as evidenced by the low amount of mis-targeted modification notable with an mTDG mutated in the major SUMO acceptor lysine (mTDG-K341R) (Figure 1D, lane 3). hXRCC1 mutated in its predicted SUMO acceptor lysine (hXRCC1-K176R; unpublished information kindly provided by Roland Steinacher), however, showed no detectable SUMOylation (Figure 1E, lane 3), thus demonstrating the selectivity of pSUMO1-mediated SUMO modification.

**Figure 2:**
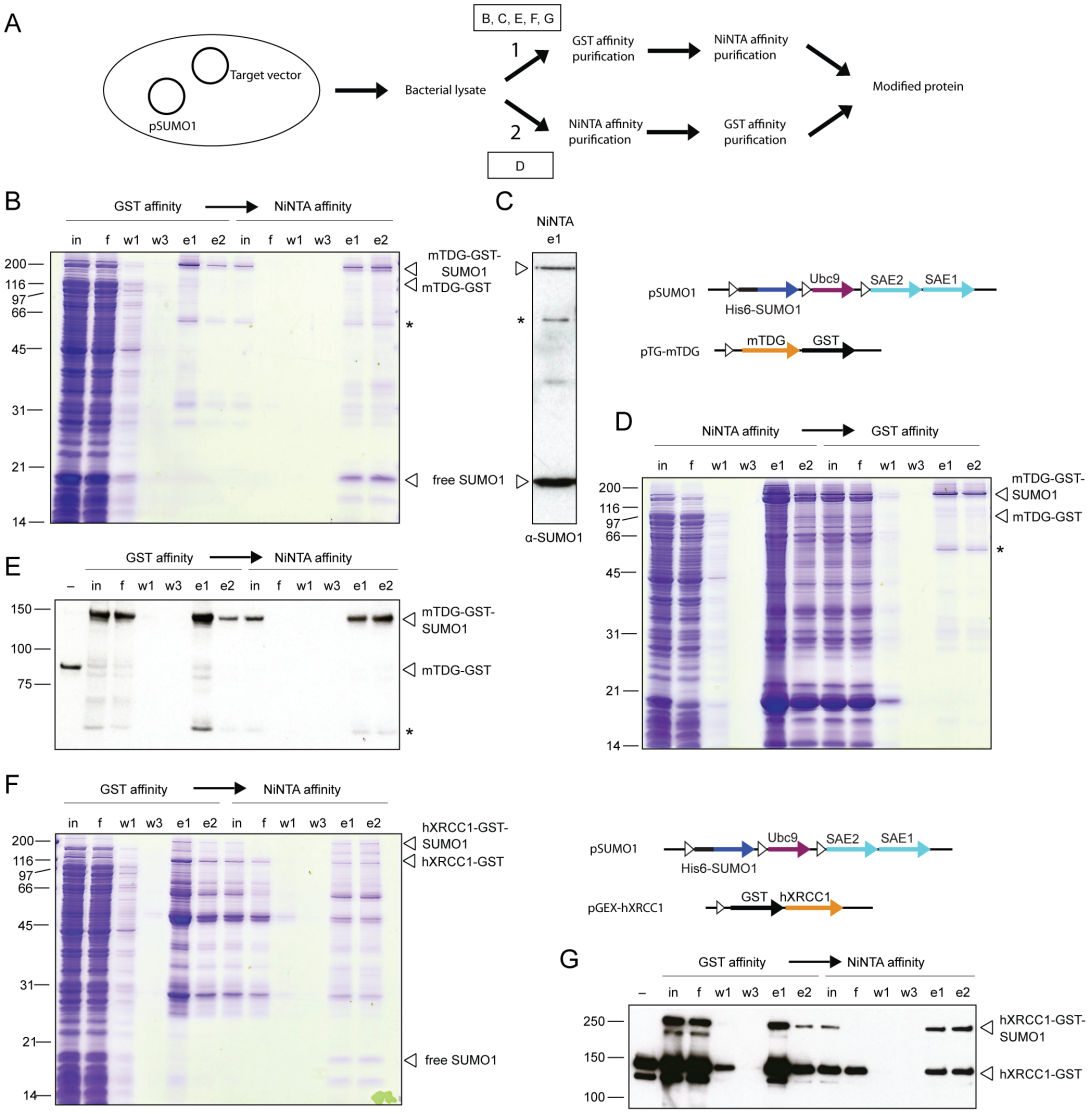
Purification of SUMO1-modified mTDG and hXRCC1 produced by in-cell SUMOylation. (**A**) Purification scheme for in-cell SUMO-modified protein. Cell lysates are subjected to subsequent GST- and Ni-NTA-affinity purification (work flow 1) or vice versa (work flow 2). Boxed letters indicate the corresponding sub-panels. Fractions of the purification of SUMO1-modified mTDG from purification work flow 1 (**B**) and 2 (**D**) and hXRCC1 from work flow 1 (**F**) were analyzed by SDS-PAGE and subsequent Coomassie blue staining and immunoblotting using monoclonal anti-GMP1 (**C**), polyclonal anti-mTDG (**E**) and anti-hXRCC1 (**G**) antibodies, respectively. in, input (cleared lysate or dialyzed elution fractions); f, flow through; w, wash steps; e, elution fractions; *, SUMO1-modified truncated mTDG.

The SUMO conjugation system presented here generates SUMOylated products with GST- and His_6_-tags fused to the target protein and the SUMO polypeptide, respectively. Purification of the modified target can thus be achieved through successive GST and Ni-NTA affinity chromatography steps (Figure 2A) as shown here for mTDG and hXRCC1. Modification of the target proteins was obtained by induced in-cell SUMOylation in *E.coli* at 25°C for 2 hours, co-transformed with pSUMO1 and pTG-mTDG or pGEX-hXRCC1. Yields of SUMO-modified mTDG and hXRCC1 proteins were estimated by stained analytic SDS-PAGE and found to be around 5 and 1.5 mg per liter bacterial culture, respectively. Thus, substantial amounts of recombinant proteins are expected to be purifiable from the in-cell SUMOylation system.

SUMOylated proteins were enriched by fractionation of the *E.coli* lysates using either sequential GST and Ni-NTA affinity chromatography (work flow 1) or vice versa (work flow 2) (Figure 2A). Following purification work flow 1, both, unmodified and modified mTDG and hXRCC1 were enriched in the elution fractions of the GST affinity column as detected by SDS-PAGE analysis followed by Coomassie staining (Figure 2B and F, GST lanes e1 and e2) and immunoblotting with anti-mTDG and anti-hXRCC1 antibodies (Figure 2E and G). Applying the pooled GST elutions to a Ni-NTA affinity column led to a further enrichment of the SUMOylated protein fractions. mTDG eluted from the column as homogeneously SUMOylated protein fraction (Figure 2B and E, Ni-NTA lanes e1 and e2). A prominent protein, however, migrating at about 20 kDa co-eluted in the main fraction and turned out to be free SUMO1 (Figure 2C). The Ni-NTA step also enriched the proportion of SUMO1-modified hXRCC1 but did not separate it entirely from unmodified hXRCC1 (Figure 2F and G, Ni-NTA lanes e1 and e2). This may be due to the propensity of hXRCC1 to dimerize through its BRCT domain under purification conditions [29], SUMO-mediated protein-protein interactions or a possible dimerization of the GST-tag, thus forming hXRCC1-SUMO1/hXRCC1 heterodimers. Following work flow 2, we observed an efficient enrichment of SUMO1-modified mTDG but also of free SUMO1 and probably some *E.coli* proteins on the Ni-NTA column (Figure 2D, Ni-NTA lanes e1 and e2). These impurities could then be separated from the SUMO1-modified target protein by GST affinity purification, which yielded homogeneously SUMOylated mTDG (Figure 2D, GST lanes e1 and e2).

### A SUMO-E2-fusion system to facilitate SUMOylation of suboptimal targets

**Figure 3:**
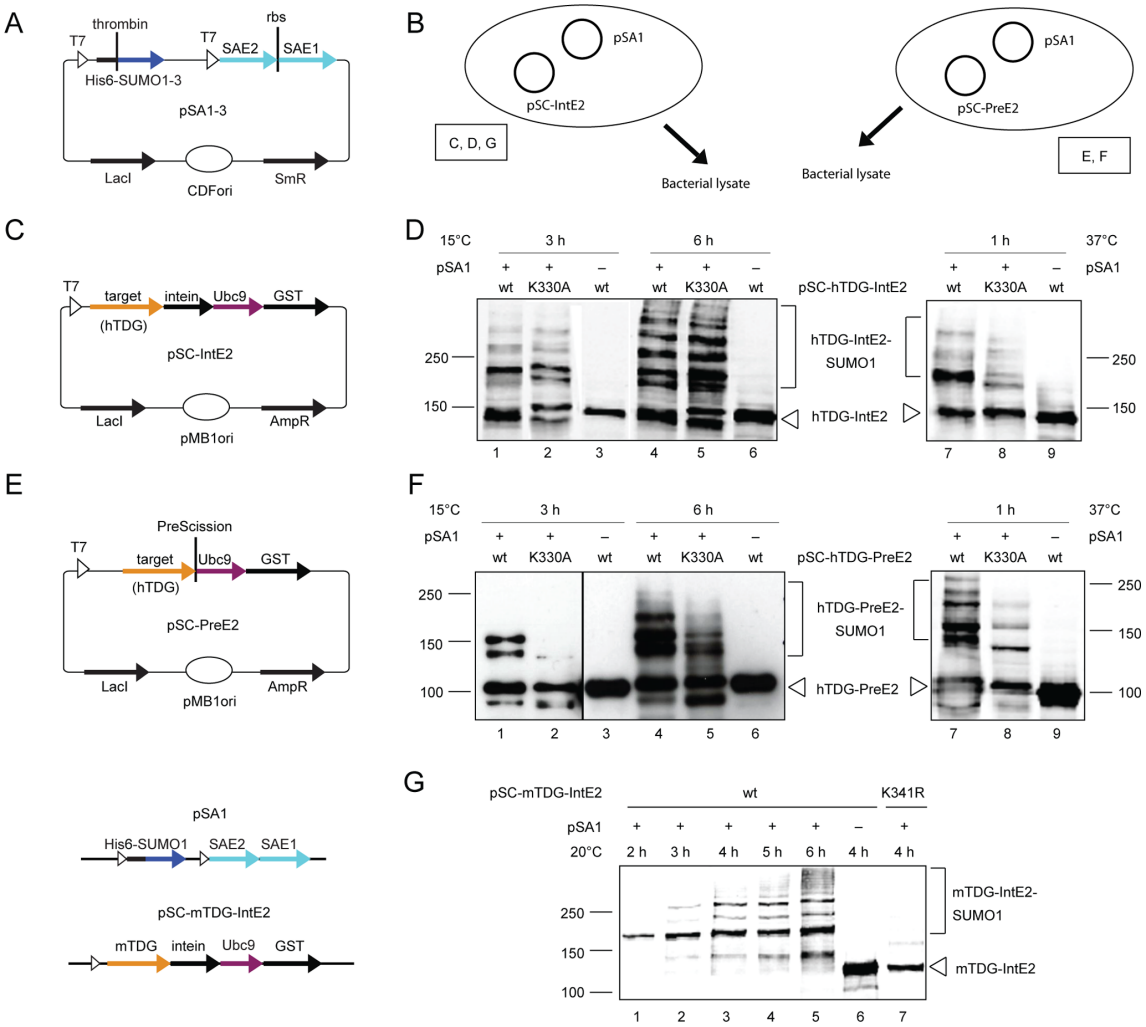
In-cell SUMOylation of TDG with the SUMO-E2-fusion system. Scheme of the SUMO-activating vectors pSA1-3, which are identical to the pSUMO1-3 vectors but lack the Ubc9 expression unit (**A**) and the Ubc9-fusion SUMO-conjugating vectors pSC-PreE2 (**E**) and pSC-IntE2 (**C**) for expression of target protein fused to a GST-tagged Ubc9 under the control of the T7 promoter. A PreScission protease cleavage site or a *Mycobacterium xenopi* GyrA intein sequence in the linker region allows for the release of the modified target from the Ubc9-GST fusion. (**B**) Experimental setup of in-cell SUMO conjugation with the SUMO-E2-fusion system. pSA1 is co-expressed with target proteins either from pSC-IntE2 or pSC-PreE2 vectors in *E.coli* BL21. Boxed letters indicate the corresponding sub-panels. Immunoblot analysis of lysates of *E.coli* cells expressing the SUMO-activating proteins (pSA1) and wild-type (wt) or SUMO acceptor site-mutated (K330A) human TDG (hTDG) from the SUMO-conjugating vectors pSC-IntE2 (**D**) or pSC-PreE2 (**F**). Expression was induced with 250 µM IPTG at 15°C for 3 and 6 h or with 1 mM IPTG at 37°C for 1 h. (**G**) SUMOylation of mouse TDG (mTDG) expressed from the pSC-IntE2 vector was followed over time by immunoblot analysis. Expression was induced with 500 µM IPTG and cells were incubated at 20°C.

Having confirmed the functionality of the humanized SUMOylation system in *E.coli*, we aimed to optimize its robustness for targets, requiring a SUMO-E3 ligase for efficient modification, by expressing the target protein as a fusion with the SUMO-conjugating enzyme [21]. To this end, we split the expression units for SUMOylation into two compatible vectors, one for SUMO activation (SA) and the other for SUMO conjugation (SC). The SUMO-activating vector (pSA1, pSA2, or pSA3) contains expression cassettes encoding mature human SUMO proteins (SUMO1-3), N-terminally fused to a His_6_-tag separated by a thrombin cleavage site, as well as both subunits of the human SUMO-activating enzyme E1 (SAE1 and SAE2) as a bi-cistronic unit (Figure 3A). The SUMO-conjugating vectors pSC-PreE2 (Figure 3E) and pSC-IntE2 (Figure 3C) were designed to express the target protein with a C-terminal fusion to the SUMO-E2 enzyme Ubc9 and the GST-tag. The inclusion of the PreScission cleavage site in pSC-PreE2 facilitates the specific release of the target proteins from the Ubc9-GST fusion by a protease digestion. The linker in pSC-IntE2 separates the SUMO target from the SUMO-E2 portion through the *Mycobacterium xenopi* GyrA intein sequence (Figure 3C) and facilitates the release of the modified target protein by self-splicing in a reducing environment [30], i.e. without protease treatment. The two constructs also provide alternative TARGET-Ubc9 configurations, should one or the other fusion cause structural constraints that compromise SUMOylation efficiency and specificity.

We first tested the functionality of the SUMO-E2-fusion system in in-cell SUMO conjugation (Figure 3B), using as targets the human TDG (hTDG), which SUMOylates *in vitro* with intermediate efficiency, its mouse ortholog mTDG as a control for high efficient SUMOylation, and hXRCC1 as an inefficiently modified substrate. We thus introduced the SUMO1-activating (pSA1) and respective SUMO TARGET-E2 vectors (pSC-hTDG-IntE2, pSC-hTDG-PreE2, pSC-mTDG-IntE2, pSC-hXRCC1-IntE2, pSC-hXRCC1-PreE2) into *E.coli* BL21(DE3). Protein expression was then induced with 250 µM and 1 mM IPTG at 15°C for 3 and 6 hours and 37°C for 1 hour, respectively. In the control reactions without SUMO activation, the non-modified full-length hTDG-IntE2 and hTDG-PreE2 fusion proteins appeared as a prominent bands migrating just below 150 kDa and around 100 kDa, respectively (Figure 3D and F, lanes 3, 6 and 9). After 3 hours of co-expression at 15°C, prominent slower migrating polypeptides appeared in cells expressing hTDG-IntE2 or hTDG-PreE2, corresponding to the SUMO1-modified hTDG fusion proteins (Figure 3D and F, lane 1). Similarly efficient and specific modification of hTDG-IntE2 or -PreE2 occurred when co-expression was done for 1 hour at 37°C under strong IPTG induction (Figure 3D and F, lanes 7 and 8). After prolonged expression for 6 hours at 15°C, additional high molecular weight SUMO modification products became apparent (Figure 3D and F, lane 4). We interpret these to represent hTDG isoforms with multiple SUMO chains or poly-SUMO chains attached, most of which do not form at the major acceptor site and are not normally seen with endogenous hTDG. The majority of these SUMO conjugates also appeared for the hTDG-K330A variant, which is mutated in the major SUMO-acceptor site (Figure 3D and F, lanes 5), hence reflecting mis-targeted modifications at either the hTDG or the fused (intein-)Ubc9-GST polypeptides, which are known and predicted SUMOylation targets. The generation of SUMO conjugates also appears to be influenced by the configuration of the TARGET-Ubc9 construct as, generally, the hTDG-IntE2 fusion generated more heterogeneity in modification products than the hTDG-PreE2 fusion, most likely because of various modifications in the intein-Ubc9-GST moiety. As with the pSUMO system (Figure 1), in-cell modification of mTDG expressed from the pSC-IntE2 vector was highly efficient, with close to 100% yields of SUMOylated mTDG (Figure 3G, lanes 1–5). At early time-points of induced protein expression (500 µM IPTG, 20°C), this setup produced a single prominent SUMOylated form of mTDG-IntE2. Only upon prolonged expression, we observe slower migrating multi-SUMOylated mTDG. As only little non-specific SUMOylation occurred with an mTDG mutated in the SUMO acceptor site (K341R) (Figure 3G, lane 7), we interpret the slower migrating mTDG-SUMO1 products to predominantly represent SUMO chain formation.

### In-extract SUMOylation increases efficiency and flexibility of the SUMO-E2-fusion system

**Figure 4:**
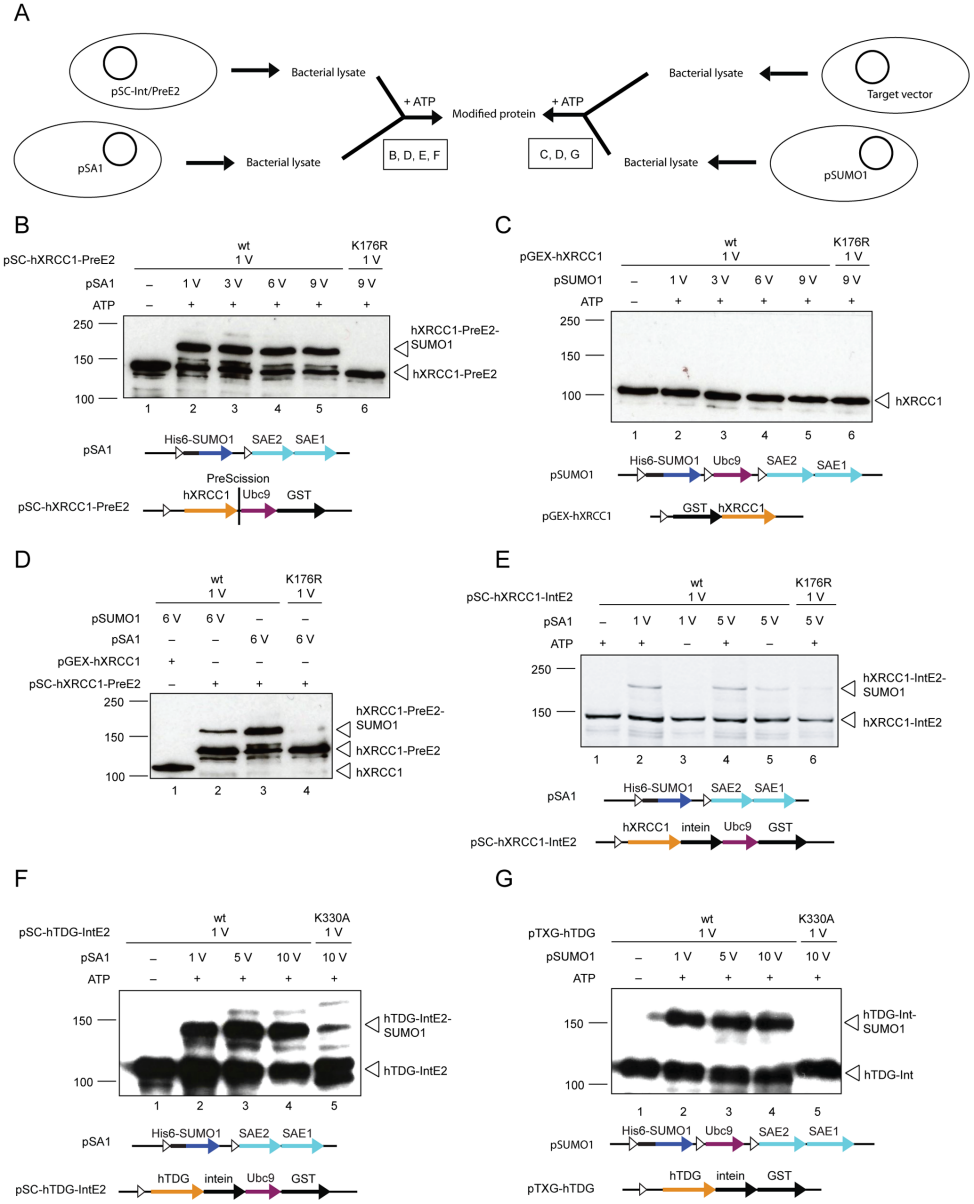
The SUMO-E2-fusion system allows SUMOylation of targets in crude cell extracts. (**A**) In-extract SUMOylation procedure using lysate from pSUMO1- or pSA1-expressing bacteria. Boxed letters indicate the corresponding sub-panels. In-extract SUMOylation efficiency with or without the addition ATP to a final concentration of 5 mM (30°C for 1 h) was assessed by immunoblot analysis. Extracts from *E.coli* BL21(DE3) cells expressing the SA1 (**B**) or the SUMO1 (**C**) system (250 µM IPTG at 30°C for 3 h) were mixed with extracts from cells expressing the fusion of Ubc9 to wild-type hXRCC1 (wt) or hXRCC1-K176R from the pSC-PreE2 plasmid (250 µM IPTG at 25°C for 3 h) with the indicated volume (V) ratio. (**D**) Direct comparison of the SUMOylation efficiency of hXRCC1-PreE2 and hXRCC1 not fused to Ubc9 with either the SA1 or SUMO1 extracts. (**E**) Crude *E.coli* BL21(DE3) cell extracts expressing wild-type (wt) or SUMO acceptor site-mutated hXRCC1 (K176R) (250 µM IPTG at 25°C for 3 h) from the pSC-IntE2 plasmids were mixed with extracts with the SA system (250 µM IPTG at 30°C for 3 h) at the indicated volume (V) ratio. Applying the same experimental conditions as above, the SUMOylation of wild-type (wt) and the SUMOylation-deficient (K330A) hTDG mutant was analyzed comparing co-incubation of extracts from *E.coli* BL21(DE3) cells expressing pSA1 and the TARGET-IntE2-fusion (**F**) or pSUMO1 and the non-Ubc9 fusion (**G**).

While in-cell SUMOylation with the SUMO-E2-fusion system was highly efficient for both TDGs, the outcome was not satisfactory for hXRCC1 (data not shown), due to inefficient expression of the fusion protein. To work around such constraints, we resorted to a strategy of expressing pSA1 and pSC-IntE2/-PreE2 vectors separately in *E.coli* cells and performing SUMO modification of target proteins in crude *E.coli* extracts without prior purification of the necessary SUMOylation factors (Figure 4A).

To assess the potential of in-extract SUMOylation, we expressed hXRCC1 from pSC-PreE2 or pSC-IntE2 and the SUMO activating factors from pSA1 in separate *E.coli* cultures at 25°C and 30°C, respectively, for 3 hours. The crude lysates of these cultures were then mixed at variable volume ratios and incubated at 30°C in the presence of 5 mM ATP for 1 hour. We then compared the efficiency of in-extract SUMOylation of hXRCC1 by the SUMO-E2-fusion system with that of extracts from cells expressing GST-tagged hXRCC1 (pGEX-hXRCC1) and the pSUMO1 system (Figure 4B and C). The SUMO-E2-fusion system produced a substantial amount of SUMO1-modified hXRCC1-PreE2 protein, which was fully dependent on an intact SUMO acceptor site (Figure 4B). By contrast, when hXRCC1 was provided without the Ubc9 fusion, in-extract SUMOylation was not detectable, even in the presence of an excess of extract providing the complete SUMOylation components (Figure 4C). Although this can be explained partly by a reduced SUMOylation capacity of the pSUMO1 lysate compared to the pSA1 lysate (Figure 4D, compare lane 2 and 3), these results show that the SUMO-E2-fusion system facilitates efficient in-extract SUMOylation of a suboptimal SUMO target like hXRCC1 (Figure 4D, compare lane 1 and 2).

To assess the impact of the configuration of the TARGET-Ubc9 fusion, we also performed in-extract SUMOylation with hXRCC1 and hTDG expressed from the pSC-IntE2 plasmid. We thus prepared crude lysates from *E.coli* expressing the pSA1 components (30°C, 3 hours induction) and the TARGET-IntE2 fusion (25°C, 3 hours induction) and incubated mixed extracts at different volume ratios at 30°C for 1 hour. This produced an appreciable amount of SUMO1-modified hXRCC1-IntE2 fusion protein (Figure 4E), largely in an ATP-dependent manner. The residual SUMO conjugation, notable without addition of ATP, at a 5-fold excess of activating over conjugating lysates (Figure 4E, lane 5) most likely reflects the pre-existence of a small amount of activated SUMO1 in the extracts. Compared to the hXRCC1-PreE2 fusion, however, the in-extract SUMOylation of hXRCC1-IntE2 fusion appeared to be less efficient; the maximum yield of SUMOylated product was generally lower for hXRCC1-IntE2 than for the hXRCC1-PreE2 fusion. Consistent with the observations from in-cell modification, in-extract SUMOylation of the TARGET-IntE2 fusions may also be less specific than that of the TARGET-PreE2 fusions, as some residual SUMO modification of hXRCC1 and hTDG mutated in the main SUMO acceptor sites appeared in the presence of an excess of SUMO-activating lysate (Figure 4E, lane 6; and Figure 4F, lane 5). These results indicate that a fusion of the SUMO-E2 enzyme to the target protein can substantially enhance in-extract SUMOylation efficiency and may be useful to compensate rate limitations due to the lack of a proper SUMO-E3 ligase in the recombinant system. Notably, the stimulatory effect of the Ubc9 fusion was less pronounced for hTDG (Figure 4F and G); in-extract SUMOylation with either IntE2-fused or non-fused hTDG generated approximately 50% modified hTDG protein with some tendency to mis-targeted modification. This is consistent with TDG's high propensity of SUMO modification in the absence of an E3 ligase and may reflect a high affinity of hTDG for SUMO1-loaded Ubc9.

Altogether, these results show that efficient SUMOylation of hTDG and hXRCC1 can be achieved with the pSA- and pSC-based SUMO-E2-fusion system. In-cell modification experiments resulted in high SUMOylation efficiency for either of the hTDG-Ubc9 fusions, but also generated considerable amounts of mis-targeted modification, either in the target protein itself or in the SUMO-E2-fusion-tag. For this particular target, stronger induction of expression at higher temperature for shorter times markedly improved the SUMOylation specificity without affecting overall protein levels. Hence, induction conditions have a strong influence on in-cell SUMOylation efficiency and specificity and thus provide opportunities for target-specific fine-tuning of the system. Overall, the PreE2 fusions seem to SUMOylate more efficiently than the IntE2 fusions. Attempts to do in-cell SUMOylation of hXRCC1 indicated that the co-expression of larger Ubc9 fusions with all SUMO components in *E.coli* may not yield satisfactory results. In such cases, SUMO modification in mixed extracts provides a valuable alternative.

### In-cell modified mTDG is biochemically active

**Figure 5:**
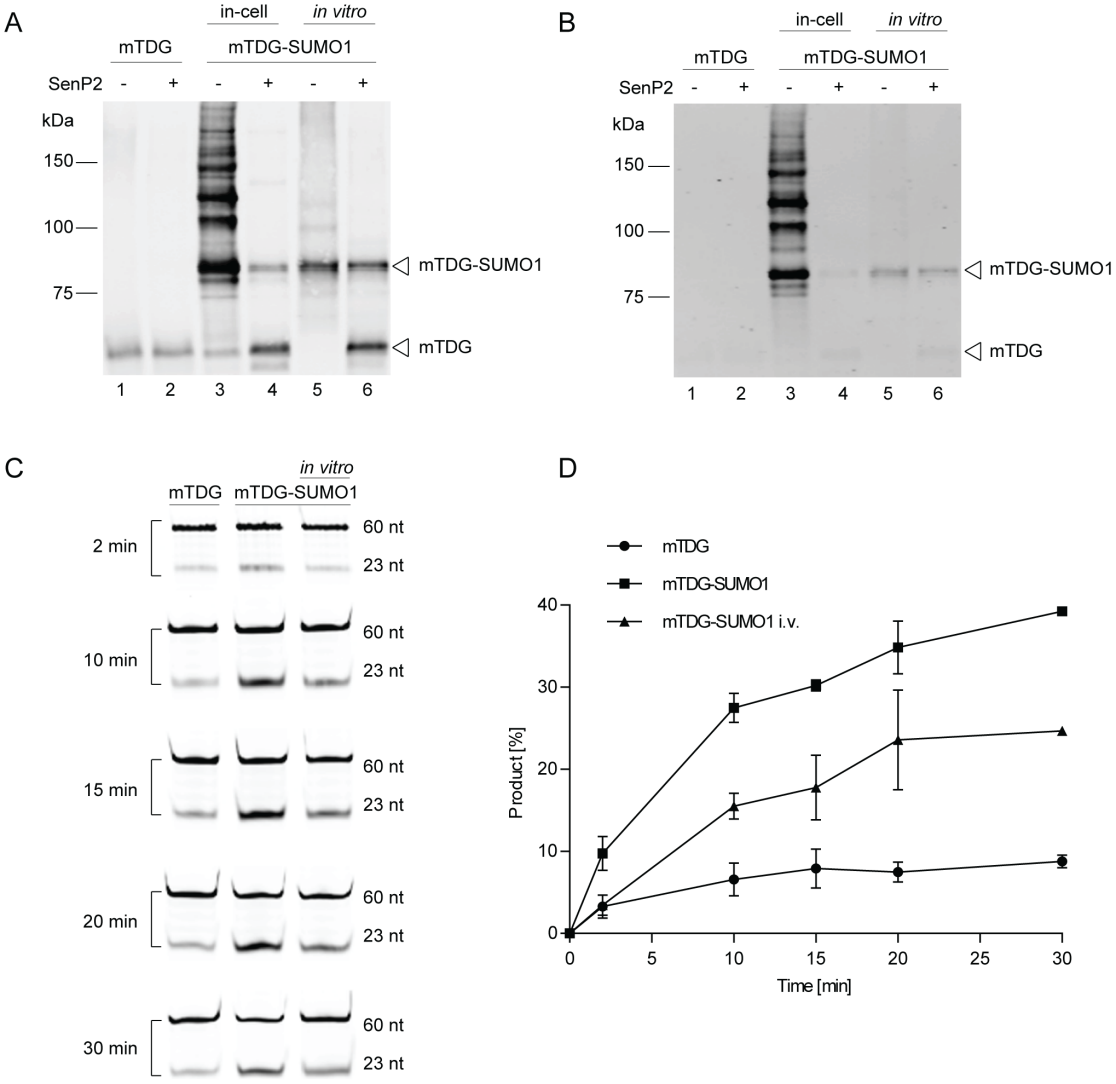
In-cell SUMOylated TDG is active and shows enzymatic turnover. 200 ng of purified mouse TDG (mTDG), purified in-cell SUMOylated mTDG and *in vitro* SUMOylated samples were analyzed by immunoblot analysis using anti-mTDG (**A**) as well as anti-SUMO1 (**B**) antibodies. Conjugated SUMO1 was cleaved with the recombinant SUMO protease SenP2 at RT for 30 min (lanes 2, 4, 6) and compared to untreated samples (lanes 1, 3, 5). (**C**) Enzymatic activity and turnover of unmodified TDG (mTDG), *in vitro* (mTDG-SUMO1 i.v.) and purified in-cell SUMOylated TDG (mTDG-SUMO1) were assessed by the base release assay with a 10-fold molar excess of G·U mismatched oligonucleotides over enzyme. Samples were taken at the indicated time-points and the relative amounts of processed 23 nucleotide product (23 nt) versus unprocessed 60 nucleotide substrate (60 nt) was quantified and depicted in (**D**). Error bars, SEM of 2 experiments.

To demonstrate that the SUMO-E2-fusion system produces authentically modified target protein, we performed in-cell modification with mTDG expressed from the pSC-IntE2 plasmid and purified the modified protein through consecutive enrichment over GST and Ni-NTA affinity columns. To test the suitability of the intein linker sequence for on-column release of the Ubc9-GST moiety, we eluted the SUMO-conjugated mTDG from the GST matrix by induced intein self-splicing in presence of 50 mM DTT. We then compared the eluted mTDG-SUMO1 with purified recombinant mTDG, either unmodified or SUMO1-modified in a defined *in vitro* SUMOylation system by immunoblot analysis using anti-mTDG (Figure 5A) and anti-SUMO1 antibodies (Figure 5B). Both antibodies detected in-cell modified mTDG as a prominent protein band migrating at around 80 kDa (Figure 5A and B, lane 3 and 5). A few higher molecular weight mTDG-SUMO1 species were also apparent, as expected for the very efficiently modified mTDG, while unmodified mTDG was hardly detectable (Figure 5A, lane 3). Upon treatment with the recombinant SUMO protease SenP2, SUMO1 was cleaved from the in-cell as well as from the *in vitro* SUMOylated mTDG (Figure 5A and B, lane 4 and 6) to generate the unmodified isoform, indicating that the detected high molecular bands are indeed SUMO1-modified mTDG protein.

To confirm that the enriched SUMO1-modified mTDG shows previously described modification-induced enzymatic features [31], we analyzed purified recombinant mTDG (Figure 5A and B, lane 1), purified in-cell SUMOylated mTDG (Figure 5A and B, lane 3) and *in vitro* SUMOylated mTDG (Figure 5A and B, lane 5) for their capacity to release uracil from G·U mismatched DNA substrates in a base release assay (Figure 5C and D). A 10-fold excess of DNA substrate over enzyme was provided in all reactions to allow an assessment of enzymatic turnover induced by mTDG SUMOylation. For unmodified mTDG, substrate processing plateaued at a product-enzyme ratio of about 2, in agreement with previous reports, showing that unmodified TDG has a strong affinity to the product AP-site and, therefore, shows the kinetics of product inhibition [24], [32]. By contrast, in-cell SUMOylated mTDG processed up to an 8-fold excess of substrate without reaching a plateau after 30 min, even more efficiently than *in vitro* SUMOylated mTDG. These findings are in line with the previously established effect of mTDG SUMOylation on the turnover rate of mTDG in a base release assay with of G·U mismatched DNA substrate. We therefore conclude that the purification of SUMO1-modified mTDG generated from the enhanced SUMO-E2-fusion system, using vectors pSA1 and pSC-mTDG-IntE2, yields functionally intact protein with the expected biochemical properties.

## Discussion

**Figure 6:**
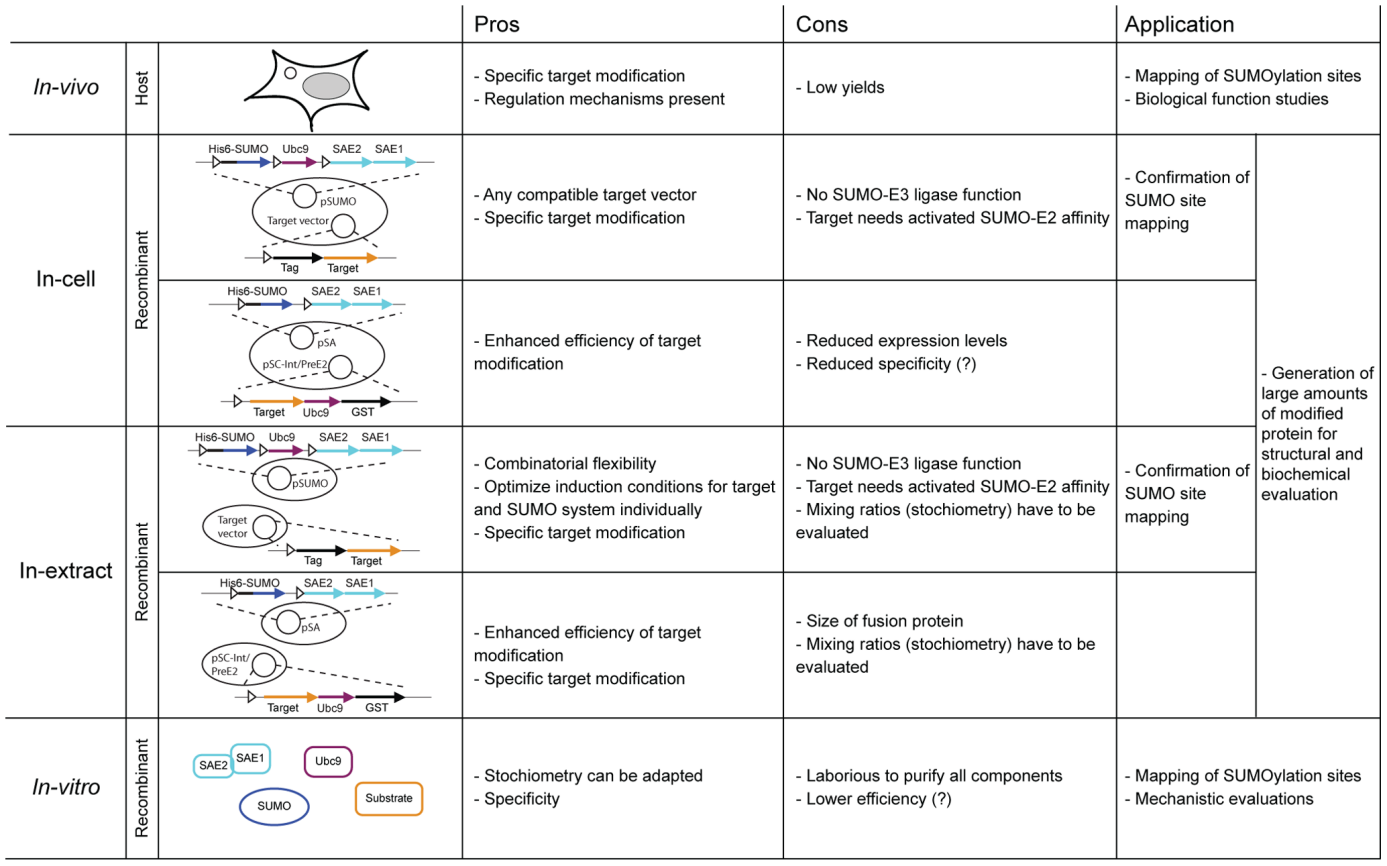
Summarizing table. Overview on our newly introduced SUMOylation systems indicating advantages, disadvantages and putative applications in comparison to host *in vivo* and purely *in vitro* systems.

Over the past decade, posttranslational protein modification by SUMO polypeptides has emerged as a key regulatory mechanism of important cellular processes [2], [5], [8], [33], [34]. The number of known and suspected SUMO targets is increasing rapidly, many of which being identified by large-scale proteomic or by bioinformatic approaches. However, insight into mechanisms underlying SUMO-regulated biological transactions is lagging behind due to the difficulty to produce recombinant SUMO-modified proteins of sufficient homogeneity and quality for biochemical analyses. To this end, we developed humanized SUMOylation systems and strategies for simple purification of modified proteins from *E.coli* (Figure 6). The pSUMO-based system expresses all essential components for SUMO modification from a single plasmid (pSUMO1-3), which is compatible with the commonly used bacterial expression vectors carrying a target protein of interest with a suitable affinity tag for purification. The SUMO-E2-fusion system is composed of a SUMO-activating vector (pSA1-3) and a SUMO-conjugating vector (pSC), containing a TARGET-Ubc9-GST fusion expression cassette. This system is suitable for both, in-cell and in-extract SUMOylation, depending on the requirements of a target protein and, the latter offering useful combinatorial options.

Unlike previously introduced SUMOylation systems [17]–[19], [35], the ones presented here consist of human components only and combine two different affinity-tags on the SUMO polypeptide and the target protein to facilitate separation of modified and unmodified target protein. The expression of the SUMO-activating enzyme E1 (SAE1 and SAE2) as separate subunits from a cistronic expression cassette avoids the SAE1-SAE2 fusion, which was shown to reduce E1 activity [20]. Consistently, SUMOylated mTDG and hXRCC1 protein appeared faster and at higher levels with our *E.coli* SUMOylation setup when compared to the system by Saitoh and colleagues [20].

SUMO-E3 ligases strongly enhance the SUMOylation efficiency and specificity in the *in vivo* situation by promoting proximity between the substrate and the SUMO-loaded Ubc9. Accordingly, more efficient and specific SUMOylation of target proteins were reported for an *E.coli* SUMOylation system when the respective E3 ligases were co-expressed [35]. However, SUMO-E3 ligases for many proteins are not known and the additional expression of such a component is likely to reduce the overall production of recombinant proteins, and is thus not suitable as a universal strategy. Fusion of Ubc9 with SUMO1 or target proteins provides an alternative [36] and has been successfully applied in mammalian cells to compensate rate limiting SUMO-E3 activity [21]. The engineered proximity makes the SUMOylation independent of the affinity between target protein and activated SUMO-E2, which is a determinant for modification efficiency [25], [37]. This TARGET-Ubc9 fusion approach is part of our *E.coli* SUMO-E2-fusion system for in-cell and in-extract modification. Using this system, we observed a fast and efficient SUMOylation of recalcitrant targets such as hXRCC1, which was only inefficiently modified without the Ubc9 fusion. Notably, canonical SUMOylation of Ubc9 itself was suggested to have a regulatory function in target discrimination, in particular for target proteins with a high affinity to SUMO [38], [39], and to stimulate the formation of SUMO chains [40]. This might explain the multiple SUMOylation events observed with the SUMO-E2-fusion system, particularly pronounced upon prolonged time of induction. The Ubc9-fusion might not only facilitate the modification of targets but could also enhance SUMOylation of the fusion-tag itself, especially when the SUMOylation site in the target is not available, i.e. mutated. However, the specificity and efficiency of target protein SUMOylation appears to be little affected by these unscheduled SUMOylation events and they will be eliminated in the course of protein purification by cleaving off the target from the fusion-tag. As authentic regulatory mechanisms are lacking and, hence, target site selection may be biased to some extent in recombinant *E.coli* SUMOylation systems, caution should be applied if the SUMO-E2-fusion is used for the validation of SUMO acceptor sites (Figure 6). In any case, the conditions for in-cell and in-extract SUMOylation with our vector systems need to be carefully evaluated and controlled so that off-target SUMOylation or non-canonical SUMO-chain formation not observed in the authentic host system can be avoided.

Based on our experience with the SUMO targets TDG and XRCC1, we can provide some basic guidelines for how to purify SUMOylated targets, although optimal conditions can vary and, thus, have to be evaluated individually. We recommend starting with the pSUMO-based system, i.e. the co-expression of a gene of interest fused to a suitable affinity tag with the SUMOylation factors provided by plasmids pSUMO1-3. The efficiency as well as the specificity of target SUMOylation is difficult to predict and likely to depend on the abundance of soluble recombinant proteins and on the affinity of the target protein with the SUMO components. For instance, SUMO modification of minor acceptor sites or the formation of poly-SUMO chains as observed with heterologous SUMOylation systems [17]–[19] may be favored when the cellular concentration of activated Ubc9 is high. It is therefore crucial to initially determine optimal induction conditions that ensure an optimal balance between expression and specificity, i.e. produce predominantly mono-SUMOylated protein, carrying the SUMO at the authentic acceptor site. In the case of TDG, which has a well-defined SUMOylation site, either a strong and fast (1 mM IPTG) induction at a high temperature (37°C) or a mild induction (250 µM IPTG) at a lower temperature (15°C) for 3 to 4 hours gave the best results with respect to SUMOylation efficiency and specificity. Applying prolonged induction times yielded more protein but also produced unwanted multi-SUMOylation at unspecific sites.

If the pSUMO-based system does not yield satisfactory SUMOylation, the SUMO-E2-fusion system, expressing the target protein from either the pSC-IntE2 or pSC-PreE2 vector, provides an alternative strategy (Figure 6). Although the intein self-cleavage mechanism used in the pSC-IntE2 vector has the advantage that no additional protease is required to remove the Ubc9 and the affinity-tag, the rather large fusion product may negatively affect the protein yields and the complex intein structure might also have an adverse effect on SUMOylation specificity. If so, the pSC-PreE2 vector with a conventional protease cleavage site offers a valuable alternative. In our experiments, we usually observed higher expression levels, increased modification efficiencies and better site specificity with targets expressed from pSC-PreE2. We recommend applying the SUMO-E2-fusion system for in-cell SUMOylation of small and easy to express proteins that SUMOylate poorly with the pSUMO-based system because of limited affinity to the activated Ubc9 or the requirement of a SUMO-E3 ligase.

For SUMO targets that are difficult to be produced and modified by co-expression, we evaluated an alternative procedure to obtain large quantities of SUMOylated proteins by in-extract conjugation, which does not require the prior purification of the SUMOylation factors. We independently expressed the TARGET-Ubc9 fusion protein and the SA system in *E.coli* BL(21) cells and prepared cleared lysate under *in vitro* SUMOylation-proficient buffer conditions. Co-incubation of these lysates resulted in a satisfactory SUMOylation of hXRCC1. Also, our data show that the Ubc9 fusion enhances SUMOylation of hXRCC1 in mixed lysates, suggesting a successful mimicking of a SUMO-E3 ligase function missing in this context. A significant advantage of this in-extract procedure is that expression conditions of the SA components and the TARGET-Ubc9 fusion constructs can be fine-tuned individually. In the case of hXRCC1, for instance, the SA system expressed most efficiently following induction with 250 µM IPTG, 30°C for 3 hours, whereas the optimal conditions for hXRCC-Ubc9 were induction with 250 µM IPTG, 25°C for 3 hours. Hence, the possibility to fine-tune these conditions will impact on the yields and quality of SUMO-modified protein. In-extract modification may also produce good yields with the pSUMO-based SUMOylation system, although in this case a prerequisite is that the target protein has a high intrinsic affinity to SUMO1-loaded Ubc9, such as TDG. This strategy offers even higher combinatorial flexibility as existing expression vectors for potential SUMO targets can be used without having to consider plasmid replication incompatibility or selection markers (Figure 6).

Purification of GST- and His_6_-tagged protein can be carried out by applying the cell extracts onto GST and Ni-NTA affinity columns irrespective of whether SUMOylation was performed in-cell or in-extract. The sequence of affinity column purification has to be evaluated individually but should only have a minor effect on the final purity. First enriching the targets on the GST affinity column gives the advantage that protein can directly be eluted from the column via release from the GST-fusion-tag. Doing so, possible modifications of the fusion-tag are eliminated and the separation of modified and unmodified target by the subsequent Ni-NTA column is not affected by a possible GST dimerization. Yet, our experience from purifying TDG and XRCC1 was that yields were better when extracts were first fractionated on the Ni-NTA column and then on the GST column. In addition, this work flow resulted in less co-purification of free SUMO1 polypeptides; an issue that will apply to all targets harboring an intrinsic SUMO interaction domain. Also, non-covalent interaction with free SUMO but also SUMO-conjugated target protein results in the co-purification of unmodified target and may require further purification steps such as size exclusion or ion-exchange chromatography or adaptation of buffer conditions (salt concentration, detergents).

In conclusion, we present tools and strategies to generate SUMOylated proteins using versatile binary expression vector systems in protease-deficient *E.coli*. They are designed to be applied for SUMOylation-related experimentation, complementary to classical investigation in the native host or *in vitro* (see Summary Figure 6). We provide purification work flows to enrich for SUMOylated protein that retains the expected biochemical properties. Owing to its high SUMOylation efficiency, the system will be suitable for screening and testing of predicted SUMOylation targets, but also for large scale purifications of modified proteins as required for biochemical and structural studies. Depending on the target, some degree of fine-tuning of expression and modification conditions will be needed to limit non-specific SUMO-conjugation. As the vector systems are available for SUMO1, SUMO2 and SUMO3, modified proteins with the variant forms can be produced to analyze and compare SUMO-specific functional properties and consequences.
